# Structured interviews on self-regulated learning strategies of medical students in the final year of medical school

**DOI:** 10.1186/s12909-023-04607-4

**Published:** 2023-08-24

**Authors:** Laura Corazza, Sepide Shirkhani, Pascal O Berberat, Marjo Wijnen-Meijer

**Affiliations:** Technical University of Munich (TUM), School of Medicine, TUM Medical Education Center, Ismaninger Straße 22, 81675 Munich, Germany

**Keywords:** Self-regulated learning, Learning strategies, Medical students, Clerkship, Clinical context, Medical education

## Abstract

**Background:**

In the final year of medical school, the educational focus is on experiences in the clinical environment. This is where students acquire most of their practical knowledge for their future career and need to optimise their Self-Regulated Learning (SRL) strategies. Hence, the current study aims to explore which SRL strategies medical students use during their clerkships in different learning settings.

**Methods:**

Structured interviews were conducted between May 2019 and December 2020 with 43 medical students during their final year in Munich, Germany. The students were surveyed about their SRL strategies. The transcribed data were thematically analysed using the measurements Strategy Use (SU) and Strategy Frequency (SF).

**Results:**

Interview data were organized into 11 SRL strategy categories. The most used SRL strategy in general was “seeking information in the internet in form of a text” (SU: 1; SF: 2.605), with an e-learning tool; followed by “seeking social assistance from doctors” (SU: 0.977; SF: 1.884), and “seeking information in books” (SU: 0.884; SF: 1.419). There were differences in the usage of SRL in different learning contexts between female and male students. For example, 95.3% of students are “seeking social assistance from doctors” when having difficulties on the ward, but only 55.8% when they need help with written tasks (e.g. medical letter). The results show a difference in SRL usage when preparing for oral-practical (79.1% books) and written (97.7% e-learning tool) exam. However, it also appears that some students do not have SRL strategies for certain situations, mostly due to a lack of time.

**Conclusion:**

Medical students in the clinical phase are adapting their SRL strategy to the learning situation. To better support students´ SRL, it is necessary to ensure availability for their preferred resources: e-learning tool and experienced physicians as supervisors. Future research should focus on strategies to handle the limited time during clerkships.

**Supplementary Information:**

The online version contains supplementary material available at 10.1186/s12909-023-04607-4.

## Background

One famous quote of David L. Sackett (1934–2015) is: *“Half of what you’ll learn at medical school will be shown to be either dead wrong or out of date within five years of your graduation; the trouble is that nobody can tell you which half - so the important thing to learn is how to learn on your own.”* [1]. This citation of the well-known pioneer of evidence-based medicine illustrates the importance of lifelong learning for physicians. Especially, due to the rapid pace of developments in medical science, doctors will regularly be confronted with new guidelines, therapy concepts, or drugs. Therefore, a core competence of medical students for lifelong professional development are learning strategies, such as Self-Regulated Learning (SRL) or Self-Directed Learning (SDL) [2, 3].

SRL is based on the assumption that learners themselves are active participants in their learning processes. In the scientific literature there is no uniform definition of the term SRL [2, 4]. Overall, SRL refers to the modulation of affective, cognitive, and behavioral processes throughout a learning experience in order to reach a desired level of achievement [2, 4]. SRL is to be distinguished from SDL [2, 4]. Both terms are often used synonymously because of their similarities such as the two dimensions external (process and event) and internal (personality and aptitude), the four key phases (defining task; setting goals and planning; enacting strategies; monitoring and reflecting), active participation, goal directed behavior, metacognition, and intrinsic motivation [3]. The differences are on the one hand in their origins. SDL originates from adult education and is mainly practiced outside of the traditional school environment, while SRL originates from cognitive psychology and is mainly practiced in the school environment. On the other hand, SDL refers to the macro-level and involves the planning of the learning trajectory and designing of the learning environment. In comparison, SRL is a micro-level concept that includes processes within task execution. In SDL, the learner initiates the learning goal, whereas in SRL the goal is usually externally set by a teacher [3]. An effective SRL is often not an effective SDL, because the learning is imposed externally, as described. Conversely, an effective SDL must include an effective SRL. Learners need to use a variety of important SRL points to achieve self-selected goals [5, 6].

Zimmerman defined SRL strategy as follows: “actions directed at acquiring information or skill that involve agency purpose (goals), and instrumentality self-perceptions by a learner” [7]. Zimmerman developed the concept of the three-phase cyclical model of SRL to ensure that individual goals are met. The three phases: (a) forethought (before), (b) performance (during), and (c) self-reflection (after), are hypothesised to be mutually dependent. In the (a) forethought phase, a task is analysed and motivational beliefs are considered. The focus of the (b) action phase is on self-control and self-observation. While in the (c) last phase, self-judgement, and self-reflection are assessed [7]. The SRL process can be influenced by the learner and the learning environment [5].

This dynamic, cyclical view of SRL underlies two principles. First, learning is dynamic and contextual. Learners make their own decisions about how best to accomplish a particular task based on situational demands. Second, the process of SRL is goal-directed and involves the integration of several sub-processes, such as planning and self-monitoring [8]. Therefore, SRL is not a single personality trait, it is more of a specific process that needs to be adapted to a particular task or situation [9]. The model can be used during any academic or clinical task in medical education, but SRL in a clinical context is different from SRL in an academic setting. Cause, in the clinical context students cannot solely focus on their own learning goals, they are subsidiary to the provision of healthcare to patients [4].

The final year of medical school in Germany is mainly focused on workplace learning. The so called “practical year” is the final stage of medical school, after passing the second of three medical state exams. During this year, students are given a lot of responsibility and acquire most of their practical knowledge and competences in order to be able to work as a physician and enter residency. During their studies, students have gained learning experiences and developed strategies to prepare themselves for various situations. Therefore, they are expected to self-regulate their learning in their final year [2]. Nevertheless, during the practical year students are confronted with certain tasks, such as with new situations on the ward, written tasks (e.g. medical letter), preparation at home for new tasks, and preparation for the final exam. There are just a few studies which examined students in a clinical setting about their SRL [10–12]. Wynter et al. explored that medical students mainly used written notes or read textbooks to learn new material and used online or downloaded questions banks for revision. The majority of students reported using e-learning tools in addition to traditional learning tools. They indicated that the gender of students was not related to the preferred use of learning material [10]. In the scoping review of Cho et al. about SRL of medical students in the clinical environment, they identified 14 eligible full text articles, of which three studies qualitatively examined how students adapt to different clinical settings [12]. Their three major findings were: (a) As a result of students´ previous educational experience, SRL changes in the clinical learning environment [12], instead of “being educated” this environment requires experiential learning and SRL [12, 13], (b) higher level of SRL are associated with academic achievement, success in clinical skills and mental health, and (c) there are various factors, e.g. intrinsic factors or interventions to support students in their SRL development, which can support SRL in medical students [12].

Research focusing on medical students´ used SRL strategies in certain situations during their clerkship is still scarce. It is important to clarify if there is a gender difference in the use of education resources in order to avoid unintended gender bias in medical education. The present study aims to address this gap. Examine which SRL strategies are used by female and male medical students in their final year will contribute a deeper understanding of which strategies students actually use in different situations in a clinical setting. Specifically, the current study determines how medical students regulate their learning in general and for specific tasks. These results can help identifying how students can be best supported in finding their individual SRL strategy to generate their best learning outcome.

### The current study aims to answer the following research questions

(1) Which different SRL strategies do female and male medical students use during their final year to remember a special examination, to encounter problems on the ward, to get prepared for a new tertial, to approach written tasks?

(2) Which different SRL strategies are used by female and male medical students to get prepared for oral-practical and written final medical state exam?

(3) How do medical students motivate themselves to learn?

## Methods

### Context

This present study was carried out at the Technical University of Munich (TUM), Germany where medical schools last six years. The first two years include pre-clinical studies, where theory-based lectures and seminars are alternated with communication and some skills training. This stage of study is completed with the first medical state exam The third to fifth year consists of the clinical core curriculum. During this period, students learn clinical skills and clinical reasoning, and much teaching takes place in clinical practice, including bedside teaching. After that, the second medical state examination must be passed in order to enter the final year, which is called “practical year”. During the practical year, the future physicians concentrate on their clinical and practical skills and consolidate their medical competences. The practical year is structured in three tertials (each 16 weeks) with clinical rotations. Students have to complete their tertial in surgery, internal medicine and an elective subject to take their final medical state examination [14]. The final medical state exam is divided into an oral-practical and a written examination. The oral-practical examination consists of practical tasks from clinical-practical subjects, including clinical-theoretical, interdisciplinary and questions on cross-sectional areas. Whereas, the written examination covers the students’ knowledge and skills that a physician needs to work independently [14].

### Participants

The participants of the present study were TUM medical students during their final year at the university hospital “Klinikum rechts der Isar” in Munich. At the time of data collection, a total of 194 TUM students were at the hospital and completed a compulsory tertial in internal medicine or surgery. For our interviews, we excluded all external students at the hospital, and all TUM students who took their compulsory tertial in other hospitals, or their elective tertial. The participants were recruited by mailing lists.

### Data collection

The students were interviewed between May 2019 and December 2020. The data collection period was long because, on the one hand, access to the clinic was limited for the interviewer due to restrictions caused by the Covid 19 pandemic. On the other hand, the number of students in the clinic was limited for the same reason, which meant that the students were involved strongly in the daily routine of the clinic. Therefore, the interviews were conducted over a longer period of time to achieve saturation.

The structured interviews were conducted and recorded by SS, and they lasted about 10 min. One face-to-face interview was conducted with each student in a one-to-one setting during the surgery or internal medicine tertial. At the time of the survey SS was a medical student and the practical year students were about one to one and a half clinical years ahead of her in their studies. The interviewees openly shared their learning strategies, knowing that SS would face the same situation in the near future.

Data collection was carried out with the help of the Self-Regulated Interview Schedule developed by Zimmerman and Martinez-Pons [7]. The interview questions, which were originally designed for high-school students, were modified accordingly for medical students in the clinical context. The interview guide was oriented to the following five learning contexts:

1. Specific learning methods.
2. Problem solving in case of difficulties on the ward.
3. Specific preparation methods at home.
4. Strategies for written tasks.
5. Learning methods for examination preparation (oral-practical/written).

For each learning context, students were asked to indicate the strategies they use during their final year. Although some of the interviews took place during the Covid 19 pandemic regulations, the impact of the pandemic was not discussed in the interviews.

SRL models emphasis the importance of integrating motivational and cognitive components of learning [15]. Thus, different motivational beliefs can contribute to promote and sustain different aspects of SRL [15]. Therefore, the focus of our last interview question was on students´ self-motivation to learn.

### Data analysis

The interviews were transcribed by SS and coded in Excel independently by SS and a second medical student to assess consistency. The students’ answers were assigned by SS and the second medical student to ten categories for learning strategies, according to Zimmerman and Martinez-Pons [7]. The categories used for the analysis were: (a) Organising and transforming, (b) Seeking information in books, (c) Seeking information in the internet in form of a text, (d) Seeking information in the internet in form of videos, (e) Keeping records and monitoring, (f) Environmental structuring, (g) Seeking social assistance from doctors, (h) Seeking social assistance from other students, (i) Reviewing records (rereading tests), (j) Reviewing records (rereading notes), (k) Rehearsing and memorising. In addition, a category labeled “Other” (l) was included for strategies that could not be assigned to any of the categories a-k.

To quantify the categories of self-regulation the authors used two measures of Zimmerman and Martinez-Pons. The first measure is called “Strategy Use” (SU), which was used to assess the SRL strategies dichotomously. More comprehensive is the measure “Strategy Frequency” (SF), which consists of the number of times a particular strategy was mentioned in the interviews, divided by the number of interviewees [7]. After coding and assigning the interviews. The calculations for the SU und SF were made in Excel.

### Reflexivity statement

The researchers in this project have backgrounds as medical educator (MWM, PB), medical education researcher (MWM, PB, LC), medical student (SS) and/or medical doctor (PB). SS conducted the interviews and conceptualised this study for her doctoral thesis. During the interviews she was a medical student in her mid-twenties as most of the research participants, but in another period of training. The interviewees had no personal relationship to SS; so they could be honest about their methods without the pressure of being judged or compared. SS was therefore familiar with the challenges of the medical exams and practical year. Additionally, through her background SS used most of the learning strategies herself. Her personal experiences were useful for the design of the research project and the interpretation of the data. LC, PB and MWM contributed to the quality of the study by means of feedback on the development of the questionnaire and iterative checks of the data analysis carried out by SS.

### Ethical approval

Approval for the study was obtained from the Ethics Committee of the Faculty of Medicine of the Technical University of Munich, application number 213/19 S. The researcher had no prior personal relationship with the students. Before the interview started, the students received information on the nature, purpose and procedure of the study, as well as their right to withhold or revoke their consent at any time. The students were notified that their participation in this study was voluntary and anonymity was assured. Informed consent was obtained from all participants.

## Results

For this study 43 medical final year students, 23 female (53.5%) and 20 male (46.5%) students, were recruited by mailing lists of students in their practical year. Out of 194 TUM students in total, this represents 22% of students during their internal medicine or surgery tertial. The student responses were mapped into categories by SS and an independent medical student. Interviewee responses were so clear that the raters assigned responses to the same category in 100% of the cases. This resulted in the very high consistency of the results.

### Organisation of SRL in general

Participants indicated their SRL strategies in all learning contexts queried. This results in SU and SF of all SRL strategies, which are presented in Table 1. The SU varies between zero and one, depending on whether a strategy was mentioned at any time during the interview or not. SU means ranged between 1 (seeking information in the internet in form of a text) and 0.023 (environmental structuring). In other words, every participant has mentioned the SRL strategy using texts from the internet, and only one participant uses the SRL strategy environmental structuring.

**Table 1:** Strategies used by all students, ordered by usage and frequency

	Strategies	All participants (n = 43)	
		SU*	SF*
1	Seeking information in the internet in form of a text	1	2.605
2	Seeking social assistance from doctors	0.977	1.884
3	Seeking information in books	0.884	1.419
4	Keeping records and monitoring	0.605	0.721
5	Seeking social assistance from other students	0.535	0.744
6	Seeking information in the internet in form of videos	0.465	0.651
7	Rehearsing and memorising	0.395	0.488
8	Rereading tests	0.302	0.302
9	Rereading notes	0.140	0.186
10	Organising and transforming	0.140	0.140
11	Environmental structuring	0.023	0.023

*Note: SU = Strategy Use; SF = Strategy Frequency

The SF measure ranged from the SU minimal level to the total number of times a strategy was mentioned during the interview. The means of SF ranged between 2.605 (seeking information in the internet in form of a text) and 0.023 (environmental structuring). In simple terms, the first SRL strategy is on average used in more than two learning contexts by every participant and the last SRL strategy is used by one participant in one learning context.

The strategy all participants (n: 43) used is “seeking information in the internet in form of a text” (SU: 1, SF: 2.605), e.g. e-learning tool. This strategy is followed by “seeking social assistance from doctors” (SU: 0.977, SF: 1.884) and “seeking information in books” (SU: 0.884, SF: 1.419). The data shows that strategies such as “environmental structuring” (SU: 0.023, SF: 0.023) e.g. “locked at home for weeks to study with fellow student”, “organising and transforming” (SU: 0.140, SF: 0.140) e.g. “create diagrams and tables”, as well as “rereading notes” (SU: 0.140, SF: 0.186) are less used by students to organise their SRL.

### Gender differences in the use of SRL in general

In order to find out whether the gender of the students has an influence on the use and frequency of strategies, the answers of female and male students were analysed separately. These differences of SU and SF in all examined learning contexts are shown in Table 2. The order of the three most used strategies is the same as for all students (seeking information in the internet in form of a text, seeking social assistance from doctors, seeking information in books), but they differ in their SF. All three SRL strategies are mentioned in more learning contexts in the interviews by male students.

**Table 2:** Strategies used by female and male students ordered by usage and frequency

Strategies	Female (n = 23)			Male (n = 20)		
	Rank*	*SU*	*SF*	Rank*	*SU*	*SF*
Seeking information in the internet in form of a text	1	1	2.522	1	1	2.700
Seeking social assistance from doctors	2	0.957	1.783	2	1	2
Seeking information in books	3	0.870	1.348	3	0.900	1.500
Keeping records and monitoring	5	0.522	0.609	4	0.700	0.900
**Seeking social assistance from other students**	**4**	0.609	0.826	**6**	0.450	0.650
**Seeking information in the internet in form of videos**	**7**	0.435	0.565	**5**	0.500	0.750
Rehearsing and memorising	6	0.478	0.609	7	0.300	0.350
Rereading tests	8	0.391	0.391	8	0.200	0.200
Rereading notes	9	0.217	0.304	10	0.050	0.050
Organising and transforming	10	0.130	0.130	9	0.150	0.250
Environmental structuring	11	0	0	10	0.050	0.050

*Note: There are no significant differences in SU and SF measurements between female and male students. The ranking only shows the different SU/SF in which the strategies were mentioned

The SU deviates by the SRL strategy “seeking information in the internet in form of videos”, for male students the strategy is on rank 5 (SU: 0.500, SF: 0.750) for female students it is on rank 7 (SU: 0.435, SF: 0.565). Which means, male students use and mention the strategy in different learning contexts more frequently than female students.

Also there is a slight difference by the SRL strategy “seeking social assistance from other students” while the strategy is for female students on rank 4 (SU: 0.609, SF: 0.826), it is for male students on rank 6 (SU: 0.450; SF: 0.650). In other words, female students use and mention the SRL strategy in all learning contexts more often than their male peers. All in all, these are slight gender differences of SRL strategy SU and SF among all learning contexts.

### Outcomes in different learning contexts

Interview questions 1 to 4 (compare **appendix**) were used to explore students’ SRL strategies in different learning contexts. The general outcomes change in relation to the learning context (compare Table 3). When asked about special methods to remember a procedure for a specific examination, the strategy “keeping records and monitoring” was mentioned by 17 students (39.5%), followed by 16 students (37.2%) who used the internet in form of videos. Noteworthy are five students (11.6%) who do not use any strategy in this learning context. When asked about dealing with problems on the ward, 95.3% of the students answered that they seek help from doctors. In some cases, they inform themselves in advance via the internet or peers before seeking advice from doctors. Another learning context was the preparation for the next clerkship. Therefore, about 44.2% of the students prepare with texts from the internet and 41.9% with books. Some of the students noted that they were only preparing for their elective subject and 18.6% reported, they were not preparing for the next tertial at all (e.g. due to time shortage). Additionally, the strategies for approaching written tasks, such as writing a medical letter over 50% of medical students are seeking assistance from experienced physicians or using templates and 11.6% do not have a strategy for this learning context.

**Table 3:** SRL strategies in certain learning contexts

	1. Specific learning method to remember an examination	2. Problem solving in case of difficulties on the ward	3. Specific preparation methods at home	4. Strategies for written tasks
**Seeking information in the internet in form of a text**	SU: 0.209	SU: 0.302	SU: 0.442*	N/A
**Seeking social assistance from doctors**	SU: 0.116	SU: 0.953*	N/A	SU: 0.558*
**Seeking information in books**	SU: 0.116	N/A	SU: 0.419*	N/A
**Keeping records and monitoring**	SU: 0.395*	N/A	SU: 0.023	SU: 0.070
**Seeking social assistance from other students**	N/A	SU: 0.209	SU: 0.047	SU: 0.023
**Seeking information in the internet in form of videos**	SU: 0.372*	N/A	SU:0.116	N/A
**Rehearsing and memorising**	SU: 0.302	N/A	SU: 0.023	SU: 0.023
**Rereading tests**	N/A	N/A	SU: 0.023	N/A
**Rereading notes**	SU: 0.070	N/A	N/A	N/A
**Organising and transforming**	N/A	N/A	N/A	N/A
**Environmental structuring**	N/A	N/A	N/A	N/A
**Other**	No strategy: SU:0.116	N/A	No preparation: SU: 0.186 Apps: SU: 0.023	Template: SU: 0.512* No strategy: SU: 0.116

*Note: Data in bold have the highest SU in these learning contexts

### SRL strategies for oral-practical and written final exam

In the following, students’ strategies to prepare for oral-practical or written examinations are compared. The results are summarized in Table 4. To the question: “Do you have special learning methods to prepare for *oral-practical exams*?” 79.1% of students answered that they seek information in books (e. g. books, question-answer-books, casebooks, and clinical books), 37.2% of participants are seeking social assistance from other students (e. g. study groups), followed by 20.9% of students who reread notes in form of protocols. Only one student did not know which SRL strategy to use, because there is insufficient time for preparation.

**Table 4:** SRL Strategies and examples medical students use to be prepared for the oral-practical and written exam

Oral-practical exam		Written exam	
Strategies	Examples	Strategies	Examples
(1) Seeking information in books	Books, question-answer books, casebooks, clinical books, examination books (SU: 0.791)	(1) Seeking information in the Internet in form of a text	medical online learning platform (SU: 0.977)
(2) Seeking social assistance from other students	Study groups (SU: 0.372)	(2) Keeping records and monitoring	Handwritten notes, notes, summaries (SU: 0.302)
(3) Re Reading notes	Protocols (SU: 0.209)	(3) Seeking information in books	Books (SU: 0.070); Scripts (SU: 0.070)
(4) Seeking information in the Internet in form of a text	medical online learning platform (SU:0.140)	(4) Organising and transforming	Creating/drawing diagrams, tables, pictures (SU: 0.093); MindMaps (SU: 0.023)
(5) Keeping records and monitoring	Handwritten notes, notes, own records (SU: 0.093)	(5) Seeking social assistance from other students	Study groups, lock-in and study with fellow students, (SU: 0.070)
(5) Seeking information in the internet in form of videos	Videos (SU: 0.093)	(5) Seeking information in the internet in form of videos	Videos (SU: 0.047); Visual learning (SU: 0.023)
(6) Seeking social assistance from doctors	Examination simulations (with doctors) (SU: 0.070); Teaching (SU: 0.023)	(6) Rereading tests	Crossing old questions online (SU: 0.047)
(7) Rehearsing and memorising	Talking alone (SU: 0.023); Being tested by a friend (SU: 0.023)	(7) Rehearsing and memorising	Repeating (SU: 0.023)
Other	Case studies (SU: 0.023); Examiners´ core focus (SU: 0.070); Knowledge from practical year (SU: 0.070); Very less time, no idea yet (SU: 0.023)	(8) Environmental structuring	Lock in at home (SU: 0.023)
		Other	Experiences from practical year (SU: 0.023)

Statements of students during the interviews:

*“To prepare for the oral exam, I use transcripts to identify examiners’ priorities, books and study groups.“*

*“We have a study group on WhatsApp.“*

*“I use case books and question-answer books, and exam simulations with the doctors on the ward.“*

In comparison, students mainly prepare themselves for *written exams* with information from the internet in form of texts (97.7%). Therefore, they use a medical online learning platform, which offers online learning plans with relevant examination topics. Another SRL strategy for 30.2% of students is keeping records and monitoring (e.g. handwritten notes, notes, summaries) and 14% of students are seeking information in books (e.g. books, scripts). Just one student uses the SRL strategy rehearsing and memorising (e.g. repeating) and another student uses the experience from the practical year.

Statements of students during the interviews:

“*For the written exam, I tried to draw diagrams or pictures of the things I read on [*a medical online learning platform*].“*

*“To study for the written exam, I barricaded myself at home with a classmate for weeks.“*

*“Just before the written exam, I reviewed my notes from the last few weeks.“*

### Self-motivation for students for learning

The students reported about different sources of motivation that drive them in their learning.

On the one hand, students report how important compensatory time off is for them, that they find motivation in pursuing various hobbies:

*“I find my balance and motivation in my hobbies. I like to do sports.“*

*“My balance from learning is to do sports or to go on holiday when I have more time. It’s not hard for me to stay on the ball and motivate myself.“*

On the other hand, students describe that learning motivates them to increase their level of performance. This is also reinforced by positive feedback from patients in their daily work. Two of the students put this in their own words, as follows:

*“It motivates me to see how I am getting better day by day.“*

*“My motivation is strengthened by positive feedback from the patients.“*

Not only the idea of achievement is decisive as a motivational factor, the interest in what is learned is also significant. For example, this student enjoys learning something interesting, which is how he finds his motivation:

*“When I learn something that interests me, it gives me great pleasure, so that motivates me.“*

Nevertheless, the prospect of later work is also crucial. Students want to meet the great responsibility they will have after graduation. Another motivational factor is the prospect of a later career and the expectation of a high salary.

*“I’m very motivated because I’m thinking about my big responsibility soon and I need to know everything by then.“*

*“My motivation for studying and working in the practical year is the prospect of my hopefully high salary later.“*

## Discussion

The present research was designed to identify the SRL strategies of medical students during their final year in a clinical environment in different learning contexts. By using the Self-Regulated Interview Schedule and measurements to quantify the categories of Zimmerman and Martinez-Pons [7], we found one SRL strategy every medical student uses: seeking information in the internet in form of a text.

The students’ SRL is influenced by the learning climate in hospitals, available learning opportunities, social interaction and learning goals [11]. The present study highlights the connection between the learning context and the SRL strategy used. Especially collaborative SRL strategies become more important [16] when students move on to new wards. Students might adapt SRL strategies that have worked in previous settings [17]. This is essential, because through clinical rotation students´ are regularly placed in unfamiliar teams and contexts [17]. Inconsistently, the strategies were used in our study in the learning contexts: procedures for a special exam, preparing for the next clerkship or writing a medical letter. This finding that SRL strategies differ by contexts is coherent with previous studies [4, 11, 18].

Another finding is that generally 97.7% of medical students seek social assistance from doctors during their clerkship. They mainly use this strategy for problems on the ward and for written tasks. Social support from teachers and peers can play an important role in setting students on the path of self-regulation. For example, Patrick et al. showed that students who receive regular support from their teachers and peers are also more engaged and more likely to use SRL strategies [19]. Also, Houten-Schat et al. described coaches or supervisors and peers as positive social influential factors to SRL [5]. Therefore, it is necessary to prepare supervisors for this role. The focus should be on stimulating the positive influential factors to support the students according to their individual SRL needs [4]. At the same time, negative factors must be limited. Houten-Schat et al. identified time pressure as a regularly named contextual barrier to SRL [5]. Also in the interviews conducted, it was regularly mentioned by the students not having enough time for preparation or developing a SRL strategy. The imbalance between the amount-to-learn and the time-to-learn is according to Nelson et al. inescapable [20]. Therefore, more tailored learning opportunities need to be provided so that lack of time does not negatively impact performance of students.

Additionally, the interviews revealed distinct differences in the preparation for oral-practical and written exams. Bauzon et al. examined the performance of students at exams. Strategies such as didactic lectures online, peer-to-peer tutoring, and the feeling of being prepared lead to better performance at exams [21]. While almost all our participants (98%) use information in the internet in form of a text to prepare for written exams, only 14% of students use a medical online learning platform to prepare for oral-practical exams. Peer-to-peer tutoring was not explicit mentioned in our interviews, but participants seek social assistance from other students: for the oral-practical exam these are 37.2% of students and for the written exam 7%. Almost all of our participants have indicated a SRL strategy for preparing for either the oral-practical or written exams. However, the results also show that some students do not have enough time for preparation, hence they do not have an SRL strategy so far, or they reduce learning content to the core focus of the examiner. This can lead to poorer exam results compared to prepared students.

When considering SRL strategy differences between female and male medical students, we identified ranking variations of “seeking social assistance from other students” and “seeking information in the internet in form of videos”. Social assistance from other students is used more often by female students than by their male peers, whereas videos are used earlier by male medical students. Our results show differences in the ranking. Several authors conclude that gender differences in SRL use in terms of self-perception, self-efficacy, competency beliefs, and/or self-belief are depending on the academic domain examined [22–25]. There is also disagreement in the literature on academic performance, while Bonsaksen et al. conclude that female students perform better than their male peers [26], Nabizadeh et al. cannot agree with this statement [27]. The cross sectional study of Elfakki et al. revealed that in general female undergraduate medical students are seeking for more help than their male peers [28]. However, according to our knowledge there is no data from medical students in the clinical environment showing gender differences in the use of SRL strategies, similar to our results.

Lastly, our participants reported about their self-motivation, and their motivation to perform well in assessments to raise their performance level and receive feedback on this. According to the self-determination theory founded by Deci & Ryan, motivation is described as a continuum spanning from amotivation through extrinsic motivation to intrinsic motivation [29]. Amotivation is a lack of determination, motivation, and intention to act [29] – in our findings students did not report about amotivation. Extrinsic motivational factors are outside forces which regulate behavior [29], regarding to our interviews the prospect of the professional career and the expectation of a high salary are high extrinsic motivational factors. Intrinsic motivation is autonomously regulated interest or enjoyment [29]. In this context, the interviewees mention how they improve daily and have interest in the subject they learn. Additionally, they mention the need of compensatory time off, they find their motivation to learn in pursuing hobbies. In education the intrinsic motivation in comparison to extrinsic motivation is associated with higher academic performance, higher engagement, higher persistence, and lower dropouts [29, 30]. Therefore, it is necessary to identify both, the motivational factors and SRL strategies students use for exam preparation in future research.

### Strengths and limitations

One of the strengths of this study is the fact that all interviews were conducted by a recent medical student (SS). This enabled the interviewer to relate to the participants and allowed an atmosphere where interviewees and interviewer act as equals. The phrasing of the interview questions were created with the Self-Regulated Interview Schedule by Zimmerman and Martinez-Pons [7], they were the same for all participants to ensure not to be suggestive. The evaluation of the interviews was carried out by two independent evaluators in order to exclude personal influences, and the consistency of the results was very high.

The findings generated from the structured interviews reflect the attitudes and opinions of the participants in a specific learning context during the Covid-19 pandemic. Therefore, the results cannot unrestricted be transferred to the general medical student in a clinical environment in another country, where students and their contexts may be very different. Also, the Covid-19 pandemic should be taken into account when interpreting the results. However, the results show tendencies of medical students in the clinical environment. It is evident that resources such as e-learning and support from supervisors are particularly important and these learning opportunities should be monitored and promoted. Nevertheless, the sample size of interviews was adequate for the stated purpose, accordingly a data saturation was achieved.

In general, there might be some response bias in the interviews, as interviewees could not give exact answers, instead there is a possibility that they may give answers they think are desirable. Furthermore, the Zimmerman and Martinez-Pons interview framework we chose limits the richness of information. The interview framework is very narrow and does not provide space for broader and in-depth-questions. This leaves the explanation of why students use an SRL strategy unanswered. Our study has an additional limitation, the outcome of the strategy used is not addressed. Further research needs to evaluate the applied SRL strategies to allow the simulation of positive influential factors and limitation of negative factors in the second phase of SRL cycle.

For practice, the learning strategies frequently used by students should be offered in high quality. In response to the aforementioned limited time to prepare for the oral-practical examination and the resulting lack of choice in SRL strategies, strategies need to be developed to support students. Nelson et al. suggested “desirable difficulty learning strategies”, such as retrieval practice (answering review questions), distributed practice (learning divided into several sessions spread over time), and interleaved practice (study several subjects at the same time) to ease the imbalance. But, after adopting these approaches to their curriculum, they could not find significant changes in the academic performance [20]. Therefore, further research must take place in this direction.

Another factor to consider for further research is that learning in the clinical context is a complex process and the interplay of many factors (e.g. time pressure and patient-related factors) affects the SRL process [11]. We suggest more research in the clinical setting, where students and residents are exposed to different situations and have to use individual SRL strategies.

## Conclusions

SRL is one important strategy for lifelong learning as doctors. Depending on the learning context medical students’ during their final year use different SRL strategies. Within the group, a clear tendency towards specific strategies in certain situations can be identified. In particular, e-learning tools are an important resource for students. Additionally, physicians who accompany students are essential for medical students during clerkships. Subsequently, these factors need to be provided in order to support students during their clerkships with their individual needs.

### Electronic supplementary material

Below is the link to the electronic supplementary material.

Supplementary Material 1

## Data Availability

The datasets generated and/or analysed during the current study are not publicly available due to data protection guidelines of the institution but are available from the corresponding author on reasonable request.

## List of abbreviations

SDL: Self-Directed Learning
SF: Strategy Frequency
SRL: Self-Regulated Learning
SU: Strategy Use
TUM: Technical University of Munich

## Declarations

### Guidelines

All methods were carried out in accordance with Declaration of Helsinki.
