# Supplementary material for: Structured interviews on self-regulated learning strategies of medical students in the final year of medical school
Source: BMC Med Educ. 2023 Aug 24;23:604. doi: 10.1186/s12909-023-04607-4 (PMC10464390; doi:10.1186/s12909-023-04607-4)
Supplement: Supplementary file 1 — Supplementary Material 1 [file 12909_2023_4607_MOESM1_ESM.docx]

# **APPENDIX**

**Interview questions:**

1. A doctor shows you the procedure for a special examination. Do you have special methods to learn or remember what is said?
2. Where do you go for help if you encounter problems on the ward?
3. Do you have special methods to prepare for your next practical year tertial at home?
4. Do you have special strategies for approaching written tasks, such as writing a doctor's letter?
5. Do you have special learning methods to prepare for the written and oral state exams? What differences are there in your preparation?
6. How do you motivate yourself to stay on the ball and keep learning?
